# Vasorelaxing Effect of Hydrolyzed Collagen from Salmon Skin in the Thoracic Aorta and Underlying Mechanisms

**DOI:** 10.3390/ijms27094084

**Published:** 2026-05-02

**Authors:** Pimchanok Mungmuang, Amnart Onsa-Ard, Jiraporn Tocharus, Rattapong Sungnoon, Rungusa Pantan, Krisana Nilsuwan, Soottawat Benjakul, Chainarong Tocharus

**Affiliations:** 1 Department of Anatomy, Faculty of Medicine, Chiang Mai University, Chiang Mai 50200, Thailand; pimchanok_m29@hotmail.com (P.M.); rungusa.p@gmail.com (R.P.); 2 Division of Biochemistry, School of Medical Sciences, University of Phayao, Phayao 56000, Thailand; amnart.on@up.ac.th; 3 Department of Physiology, Faculty of Medicine, Chiang Mai University, Chiang Mai 50200, Thailand; jiraporn.tocharus@cmu.ac.th (J.T.); sungnoon@gmail.com (R.S.); 4 International Center of Excellence in Seafood Science and Innovation, Faculty of Agro-Industry, Prince of Songkla University, Songkhla 90110, Thailand; krisana.n@psu.ac.th (K.N.); soottawat.b@psu.ac.th (S.B.); 5 Functional Food Research Center for Well-Being, Multidisciplinary Research Institute, Chiang Mai University, Chiang Mai 50200, Thailand

**Keywords:** hydrolyzed collagen, salmon skin, vasorelaxation, rat aorta, hypertension

## Abstract

Hydrolyzed collagen (HC) derived from salmon skin is a promising source of bioactive peptides. In this study, the vasorelaxant effects and potential mechanisms of action of HC on isolated rat thoracic aorta rings were investigated using the organ bath technique. The vasorelaxant properties of HC were evaluated using aortic rings from Wistar rats pre-contracted with phenylephrine (PE) or potassium chloride (KCl). HC induced significant vasorelaxation in both endothelium-intact and endothelium-denuded rings, indicating that its mechanism of action was independent of the endothelium and involved direct effects on vascular smooth muscle cells. The vasorelaxant effect of HC was reduced when pre-contraction was induced by tetraethylammonium chloride (TEA). However, the vasodilatory effects of HC were not significantly inhibited by all K^+^ channel blockers, including glibenclamide, barium chloride (BaCl_2_), or 4-aminopyridine (4-AP). Additionally, pre-incubation with prazosin, an α-adrenoceptor blocker, significantly reduced the vasorelaxation induced by HC, whereas propranolol, a β-adrenoceptor blocker, had no effect. In addition, HC inhibited CaCl_2_-induced contractions induced by both PE and caffeine in a Ca^2+^-free solution. Therefore, HC exhibited the vasorelaxant effects through an endothelium-independent mechanism. The vasodilatory effects of HC were associated with the activation of K_Ca_ channels, suppression of PE-induced contraction via α_1_-adrenergic receptor pathways, and inhibition of CaCl_2_-induced contractions by modulating intracellular Ca^2+^ release and extracellular Ca^2+^ influx in vascular cells.

## 1. Introduction

Elevated blood pressure is a major determinant of cardiovascular disease, playing a central role in mortality worldwide. The high frequency of hypertension rises with age, due to endothelial dysfunction and increased vascular stiffness. Peripheral vascular resistance influences the regulation of blood pressure through the control of vasoconstriction and vasodilation. Therefore, vascular dysfunction is considered a crucial therapeutic target in the management of hypertension [1,2].

Salmon skin, a byproduct of salmon processing, is rich in collagenous proteins and can be hydrolyzed to bioactive collagen peptides [3]. The bioactivities of these peptides are enhanced by their reduced molecular size, conferring increased antioxidant and antimicrobial activity, which is influenced by their molecular weight [4]. Previous studies have suggested that gelatin hydrolysate from salmon skin significantly decreases systolic blood pressure, thereby exerting antihypertensive effects in spontaneously hypertensive rats [5]. Other reports indicated that collagen extracted from Atlantic salmon skin, with molecular weights below 1 kDa, contained angiotensin-converting enzyme (ACE) inhibitory peptides that acted as functional foods with antihypertensive properties [6]. In addition, our previous studies reported that hydrolyzed collagen (HC) from salmon skin exerts antihypertensive effects by reducing oxidative stress and inflammation by decreasing proinflammatory cytokine production through attenuation of nuclear factor kappa B (NF-κB) activation. Moreover, HC improved vascular function by mitigating collagen type I accumulation and reducing matrix metalloproteinase (MMP-9) expression in Nω-nitro-L-arginine methyl ester (L-NAME)-induced hypertensive rats [7]. However, the present study focused on the ex vivo vasorelaxant effects of HC from salmon skin on isolated rat aortic rings using an organ bath technique to investigate its impact on vascular function.

## 2. Results

### 2.1. Vasorelaxant Effects of HC on Isolated Rat Aorta Activated by PE and KCl

**Figure 1:**
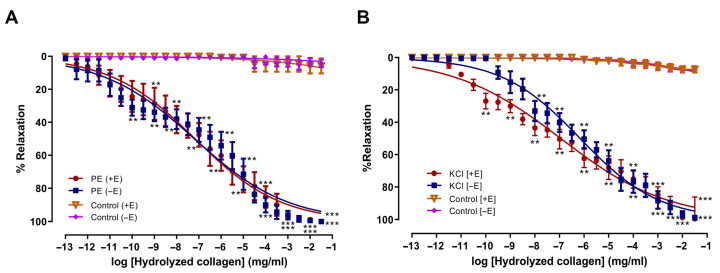
Effects of HC from salmon skin on vascular relaxation on both endothelium-intact (+E) and endothelium–denuded aortic rings E), (**A**) PE -induced (10^−5^ M), (**B**) KCl-induced (80 mM). Values presented as mean ± S.E.M. Statistical analysis was conducted using one-way ANOVA followed by Dunnett’s post hoc test. ** *p* < 0.01, *** *p* < 0.001, significant difference in comparison with control groups (absence of HC) (*n* = 6 rings per group).

HC produced vasorelaxation in PE (10 µM)–precontracted aortic rings regardless of whether the endothelium was present or removed, suggesting the effect involves receptor-operated Ca^2+^ channels (ROCCs). The EC_50_ values of HC for the endothelium-intact and endothelium-denuded rings were 10^−7^ mg/mL and 10^−7^ mg/mL, respectively. Both groups exhibited E_max_ values of 100%, compared with control values of 5.79 ± 4.51% and 4.16 ± 2.2% (*p* < 0.001) (Figure 1A). In the case of the voltage-operated calcium channels (VOCCs), activation by KCl (80 mM) demonstrated that HC attenuated KCl-induced contraction in both groups. However, there was no significant difference between endothelium-intact and endothelium-denuded rings. The vasorelaxation curves were similar for both groups. The EC_50_ values of HC were 10^−7^ mg/mL for intact rings and 10^−6^ mg/mL for denuded rings, with E_max_ values of 99.48 ± 0.53% and 98.81 ± 1.19%, compared to control values of 7.26 ± 1.83% and 7.83 ± 1.55% (*p* < 0.001), respectively (Figure 1B). These results indicate that the vasorelaxant effect of HC did not differ significantly between endothelium-intact and endothelium-denuded rings. However, in both PE- and KCl–induced contractions, HC treatment resulted in significantly greater relaxation than in controls.

### 2.2. The Effects of HC–Induced Relaxation on K^+^ Channels

**Figure 2:**
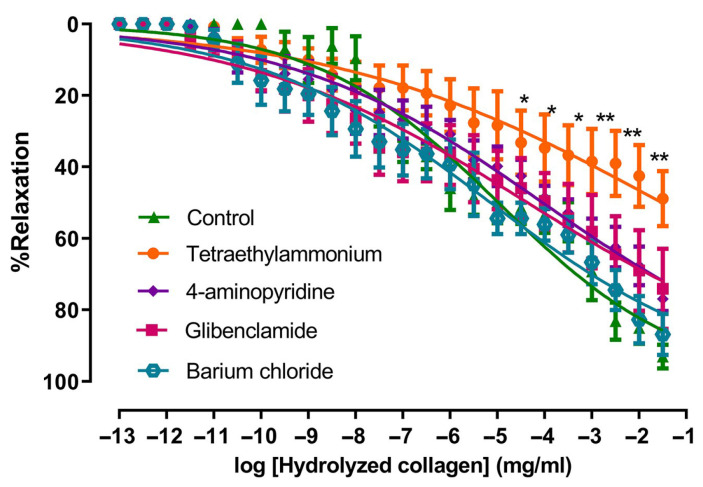
Effects of HC on individual types of potassium channels. Values presented as mean ± S.E.M. Statistical analysis was conducted using one-way ANOVA followed by Dunnett’s post hoc test. * *p* < 0.05, ** *p* < 0.01 significant difference in comparison with the control group (absence of drug) (*n* = 6 rings per group).

To evaluate the role of individual K^+^ channels in HC-evoked relaxation, endothelium-denuded aortic rings were incubated with selective K^+^ channel blockers before stimulation. In the presence of tetraethylammonium (TEA), which indicates partial involvement of TEA-sensitive K^+^ channels (including K_Ca_ channels), the vasorelaxant effect of HC was significantly reduced compared with the control group. The E_max_ value for the TEA-treated group was 48.82 ± 7.63%, which was significantly lower than that of the control group (94.03 ± 3.24%, *p* < 0.01). In contrast, the other K^+^ channel blockers, including 4-aminopyridine (K_V_ blocker), glibenclamide (K_ATP_ blocker), and barium chloride (K_ir_ blocker), did not result in significant reductions in vasorelaxation in comparison with controls. The E_max_ values for these groups were 76.68 ± 3.27%, 74.04 ±11.13%, and 86.82 ± 5.68%, respectively (Figure 2).

### 2.3. Role of Adrenergic Receptors in HC-Induced Vasorelaxation

**Figure 3:**
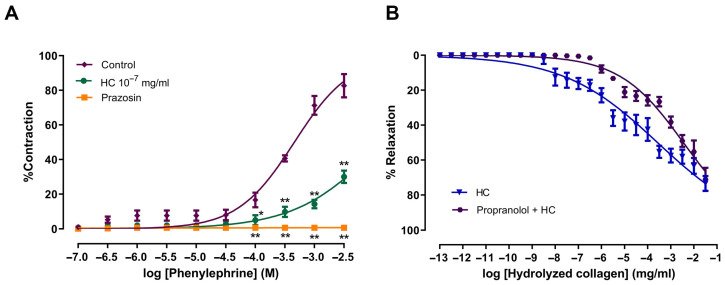
Effects of HC on relaxation in PE -induced with the effect of α-adrenoreceptor and β-adrenoreceptor in the presence or absence of (**A**) prazosin (0.1 µM) and (**B**) propranolol (1 µM). Values represent mean ± S.E.M. Statistical analysis was conducted using one-way ANOVA followed by Dunnett’s post hoc test and unpaired *t*-test. * *p* < 0.05, ** *p* < 0.01 significant difference in comparison with the control groups (absence of drug) (*n* = 6 rings per group).

The involvement of adrenergic receptors in HC-induced vasorelaxation was evaluated in the aortic rings. Pre-incubation with HC (10^−7^ mg/mL) or prazosin (0.1 µM), followed by cumulative addition of PE (1 nM to 100 µM), significantly reduced the level of PE-induced contraction when compared to the control group (*p* < 0.01). The E_max_ values for the HC and prazosin treatments were 30 ± 3.63% and 0.67 ± 0.67%, respectively, compared to 82.67 ± 6.69% in the control group (Figure 3A). Next, the vasorelaxant effect of HC in relation to the β_2_-adrenergic receptor was examined using propranolol (1 µM), a non-selective β-adrenergic receptor antagonist. The results showed that preincubation with propranolol did not significantly alter the effect of HC-induced vasorelaxation. The E_max_ values were 70.93 ± 6.52% for the propranolol-treated group and 73.34 ± 4.21% for the HC group (Figure 3B). The results suggest that HC induces vasorelaxation, which is partly mediated by α_1_-adrenergic receptors.

### 2.4. The Effects of HC on Ca^2+^ Inhibition in Isolated Aorta

**Figure 4:**
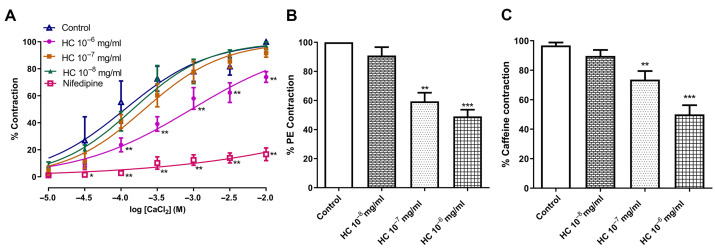
Effects of HC on the action of calcium influx (**A**) nifedipine (1 μM), calcium efflux (**B**) PE (10 μM), and (**C**) caffeine (20 mM). Values shown as mean ± S.E.M. Statistical analysis was conducted using one-way ANOVA followed by Dunnett’s post hoc test. * *p* < 0.05, ** *p* < 0.01, *** *p* < 0.001, significant difference in comparison with control groups (*n* = 6 rings per group).

To investigate whether the vasorelaxant effect of HC involves voltage-gated calcium channels, aortic rings pre-treated with HC (10^−6^ mg/mL) showed a significant decrease in CaCl_2_-induced contraction compared to the control group, with maximal contraction of 73.93 ± 3.99% versus 100% in controls (*p* < 0.01). Nifedipine (a Ca^2+^ channel blocker) significantly inhibited the contraction of 16.69 ± 4.68% induced by CaCl_2_ (*p* < 0.01). In contrast, the relaxant effect of HC (10^−7^ mg/mL and 10^−8^ mg/mL) did not differ significantly from that of the control group, with maximal contractions of 91.66 ± 3.03% and 97.14 ± 2.86%, respectively (Figure 4A). Next, the effect of HC on intracellular Ca^2+^ release from the sarcoplasmic reticulum (SR) was investigated. In endothelium-denuded rings, pretreatment with HC (10^−6^ and 10^−7^ mg/mL) significantly attenuated PE- and caffeine-induced contractions (*p* < 0.01 and *p* < 0.001, respectively) compared with the control group. The maximal contraction values of PE for HC at 10^−6^ mg/mL and 10^−7^ mg/mL were 49.03 ± 4.58% and 59.41 ± 5.92%, respectively, compared with 100% for the control group (Figure 4B). The values in the case of caffeine were 50.06 ± 66.24% and 73.60 ± 5.90% vs. 96.82 ± 2.02%, respectively (Figure 4C). Our results indicate that the vasorelaxant effect of HC may involve the inhibition of extracellular influx and intracellular efflux from SR.

## 3. Discussion

The results of this study demonstrate that HC from salmon skin induces vasorelaxation in rat aortic rings. This effect could result from disrupted Ca^2+^ channels, inhibition of Ca^2+^ release from intracellular stores, and activation of K_Ca_ channels. Additionally, the vasorelaxation effect of HC is potentially regulated through the α_1_-adrenergic receptor. Our results are consistent with our prior studies showing that HC from salmon skin attenuated hypertension and vascular dysfunction in L-NAME-induced hypertensive rats [7].

PE induces vasoconstriction by increasing extracellular calcium influx and promoting Ca^2+^ release from the SR via ROCC activation [8]. In contrast, high K^+^ concentrations depolarize the cell membrane, activating VOCC, enhancing myosin light chain (MLC) phosphorylation, and stimulating the PI3K-C2α and Rho kinase signaling pathways to promote vascular contraction [9]. Our results indicate that HC significantly inhibits both PE-induced and KCl-induced contractions, suggesting that HC acts through IP_3_-dependent signaling [10,11] and modulates the influx of Ca^2+^ in KCl-depolarized cells. Thus, in this study, the main focus is on endothelium-independent pathways. Furthermore, HC appears to activate the opening of K^+^ channels, which are essential in the regulation of vascular smooth muscle relaxation. There are four main types of K^+^ channels in vascular smooth muscle cells: specifically inward rectifier K^+^ (K_ir_), voltage-gated K^+^ (K_V_), ATP-sensitive K^+^ (K_ATP_), and Ca^2+^-activated K^+^ (K_Ca_) channels. Activation of these channels leads to membrane hyperpolarization, reducing Ca^2+^ influx and promoting vasodilation [12,13]. Our findings suggest that HC significantly attenuates vascular contraction in endothelium-denuded aortic rings, with partial involvement of TEA-sensitive K^+^ channels. However, 4-aminopyridine (K_V_ blocker), glibenclamide (K_ATP_ blocker), and barium chloride (K_ir_ blocker) did not affect HC-induced relaxation in rat aortic rings [14,15]. In relation to HC, this mechanism of HC can prevent calcium influx from outer membrane cells via K_Ca_ channels. Next, HC-induced relaxation via β_2_-adrenergic receptors located on the vascular smooth muscle cell membrane was observed. β-adrenoceptors are G protein-coupled transmembrane receptors involved in the regulation of vascular tone. The stimulation of the β-adrenergic receptors is concerned with an increase in the concentration of cAMP by activation of the adenylyl cyclase, resulting in a reduction in vascular tension. In this mechanism, blocking with propranolol (a non-selective β-adrenergic receptor blocker) did not affect the response to HC-induced relaxation [16,17]. In accordance with these results, we concluded that the role of β_2_-adrenoceptor activation did not involve vascular relaxation by HC. This study indicated that HC inhibited PE-induced contraction mediated by the α_1_-adrenergic receptor, using prazosin (α_1_-adrenergic receptor antagonist) to assess the activity of HC. The result demonstrated that HC inhibited PE-induced contraction in the same pattern as was observed in the prazosin tests [18,19]. This result suggests that the vasorelaxation effect of HC is potentially regulated through the α_1_-adrenergic receptor, as confirmed by our initial study. Therefore, the mechanism of the HC on the vasorelaxant activity involved in the inhibition of Ca^2+^ influx through VOCC and intracellular Ca^2+^ release was investigated [20]. To examine this, the aortic rings were contracted in Ca^2+^-free KCl (80 mM) to induce membrane depolarization, which drives external Ca^2+^ into the vascular smooth muscle cells [21,22,23]. This process leads to the opening of VOCCs in endothelium-denuded rings. The results showed that HC significantly inhibited Ca^2+^-induced contraction in KCl (80 mM), indicating that it suppresses extracellular calcium influx through VOCCs. Furthermore, nifedipine, an L-type VOCC antagonist with strong Ca^2+^-binding properties and the ability to induce a depolarizing shift in surface charge, completely inhibited KCl (80 mM)-induced contraction [24]. These findings suggest that HC-induced vasorelaxation with the inhibition of L-type Ca^2+^ channels, in a similar way to the effects of nifedipine. HC also appears to reduce contraction by inhibiting the release of Ca^2+^ from intracellular stores as induced by PE and caffeine. PE induces contraction by activating Ca^2+^ through activation of the specific IP_3_R on the SR, leading to Ca^2+^ release, while caffeine triggers Ca^2+^ release through the activation of the ryanodine receptors (RyR) on the SR [25]. Both mechanisms produce transient vascular smooth muscle contraction in Ca^2+^-free solution [26,27,28]. Notably, HC significantly reduces contraction effects by both PE and caffeine. These findings suggest that HC may block intracellular calcium released through both IP_3_R and RyR on the SR membrane. Consequently, this study demonstrates that HC also affects, as assessed by contractile responses in isolated rat aorta experiments. In addition, the rat aortic ring model was used to reduce vascular tone and to investigate the underlying mechanisms. These findings enable further study of the molecular regulation of endothelial dysfunction that can lead to hypertension. However, the main limitation of this study is that the effects of HC were examined only in an ex vivo isolated rat aorta model, which limits conclusions about the molecular targets and signaling pathways involved.

## 4. Materials and Methods

### 4.1. Chemicals

Phenylephrine (PE), acetylcholine chloride (ACh), propranolol hydrochloride, prazosin hydrochloride, tetraethylammonium chloride (TEA), barium chloride (BaCl_2_), glibenclamide, 4-aminopyridine (4-AP), caffeine, nifedipine, potassium chloride (KCl), dimethyl sulfoxide (DMSO), and ethylene glycol-bis (β-aminoethyl ether)-N,N,N′,N′–tetraacetic acid (EGTA) were purchased from Sigma-Aldrich (St. Louis, MO, USA). Glibenclamide and nifedipine were dissolved in DMSO. Hydrolyzed collagen (HC) and other compounds were dissolved in distilled water.

### 4.2. Preparation of Hydrolyzed Collagen from Salmon Skin

Frozen skins of sockeye salmon (*Oncorhynchus nerka*) were cut into small pieces (3.0 × 3.0 cm^2^) using an electric sawing machine. Non-collagenous protein was then removed using an alkaline solution as described by Benjakul et al. [29]. Subsequently, the prepared skin was swollen with the aid of 0.05 M citric acid (1:10, *w*/*v*). The mixture was agitated for 15 min and allowed to stand for 45 min. The swollen skins were rinsed with water until a neutral pH was reached.

HC was prepared as described by Nilsuwan et al. [30]. To the swollen skins, distilled water was mixed at a ratio of 1:5 (*w*/*v*). The mixture pH was adjusted to 8. Papain and Alcalase were added at 3% and 4% (*w*/*w*, based on solid content), respectively. Hydrolysis was conducted at 60 °C with continuous stirring for 4 h. Then, the inactivation of enzymes present in the hydrolysate was done at 90 °C for 15 min. HC solution was subsequently defatted using a disk stack centrifugal separator (SPX FLOW Technology Italia S.p.A., Milan, Italy) with the feed rate of 2.0 L/min. Defatting was carried out for 9 cycles. HC obtained was lyophilized [30], and the powder was further defatted using isopropanol at a ratio of 1: 10 (*w*/*v*) for 3 cycles. HC powder was sealed in nylon polyethylene laminated bags and stored at −20 °C.

The degree of hydrolysis, measured using the 2,4,6-trinitrobenzenesulfonic acid (TBNS) method, was 35% as described by Benjakul and Morrissey [31]. MW of peptides was determined according to the method of Benjakul et al. [32]. MALDI-TOF analysis was carried out using a MALDI/TOF mass spectrometer (Autoflex Speed, Bruker, Bremen, Germany) equipped with a 337 nm N2 laser. The flexControl software version 3.4 (Bruker Daltonik, GmbH, Bremen, Germany) was used. The peptide with MW of 1460 Da was dominant, followed by those having MW of 1975 and 1530 Da, respectively.

### 4.3. Experimental Animals

Male Wistar rats (weight, 250–300 g) were obtained from Nomura Siam International, Bangkok, Thailand. The animals were acclimated under a 12 h light/dark cycle ad libitum access to standard chow and water. All experimental procedures were conducted in accordance with protocols approved by the Institutional Animal Care and Use Committee, Faculty of Medicine, Chiang Mai University, Thailand (Permit Number: 08/2565) and were conducted in accordance with the National Institutes of Health Guide for the Care and Use of Laboratory Animals.

### 4.4. Preparation of Rat Aortic Rings

After euthanasia with isoflurane, the thoracic aorta was removed from the rats. The aortas were carefully cleaned and cut into segments approximately 3–4 mm long. Aortic rings were mounted in 2 mL organ bath chambers containing Krebs solution (mmol/L: NaCl 122.0, KCl 4.9, HEPES 10.0, KH_2_PO_4_ 0.5, NaH_2_PO_4_ 0.5, MgCl_2_ 1.0, glucose 11.0, and CaCl_2_ 1.8), maintained at pH 7.4 and 37 °C, and continuously aerated with 95% O_2_/5% CO_2_. Each ring was suspended between two stainless-steel hooks under an optimal resting tension of 1 g and allowed to equilibrate for 60–90 min. Changes in isometric tension were recorded using isometric force transducers (Iworx Systems, Inc., Dover, NH, USA) connected to a computer-based data acquisition system (LabChart 7; ADInstruments, Sydney, Australia). For endothelium-denuded preparations, the endothelial layer was gently removed by rubbing the luminal surface with a cotton swab moistened with Krebs solution. After baseline tension had stabilized, the aortic rings were challenged with phenylephrine (PE, 10 µM) to induce vasoconstriction. After the contraction plateaued, ACh was added to induce vasorelaxation. The presence of functional endothelium was confirmed when ACh induced vasorelaxation of at least 90% following pre-contraction with PE, indicating endothelium-dependent vasorelaxation. Effective removal of endothelium was demonstrated when ACh-induced vasorelaxation was less than 10%.

### 4.5. Experimental Protocol

#### 4.5.1. Vasorelaxant Effects of HC on PE- or KCl-Precontracted Aortic Rings

PE (10 µM) or high KCl (80 mM) was used to induce contraction in endothelium-intact or endothelium-denuded aortic rings. HC (10^−13^–10^−1.5^ mg/mL) was then added cumulatively to induce vasorelaxation. The vasorelaxant effect of HC was expressed as percentage relaxation relative to the maximal contraction induced by either PE or KCl, and the responses between these groups were compared.

#### 4.5.2. The Vasorelaxant Effects of HC on the K^+^ Channels

To investigate the involvement of K^+^ channels in the vasorelaxant effect of HC, aortic rings were pre-incubated with four types of K^+^ channel blockers, including non-selective calcium-activated potassium (K_Ca_) channels, non-specific adenosine triphosphate-sensitive potassium (K_ATP_) channels, voltage-dependent K^+^ (K_V_) channels, and inwardly rectifying K^+^ (K_ir_) channels. Endothelium-intact aortic rings were pre-incubated with the following inhibitors: TEA (non-selective potassium channel, partially sensitive-K_Ca_ blocker, 5 mM), glibenclamide (K_ATP_ blocker, 10 μM), 4-AP (K_V_ blocker, 1 mM), and BaCl_2_ (K_ir_ blocker, 1 mM) for 20 min before contraction was induced by PE (10 μM). The HC in cumulative concentrations (10^−13^ to 10^−1.5^ mg/mL) was added to the chamber, and the results were then compared to those of the control group (without inhibitors).

#### 4.5.3. Evaluation of the Relaxant Effects of HC on α_1_-Receptors and β-Receptors

To determine whether HC-induced relaxation involved α_1_-receptors, endothelium-denuded rings were preincubated with prazosin (0.1 µM) and HC (10^−7^ mg/mL) for 20 min before PE-induced precontraction. Contraction was then induced by the cumulative addition of PE (1 nM to 100 µM). In addition, to assess whether the vasorelaxant effect of HC was mediated by β_2_-receptors, the β-adrenoceptor antagonist propranolol (1 µM) was used. Endothelium-denuded aortic rings were pre-incubated with propranolol for 20 min before the addition of PE (10 µM). HC (10^−13^ to 10^−1.5^ mg/mL) was then added cumulatively. Percent relaxation to HC was compared in the absence and presence of prazosin and propranolol.

#### 4.5.4. The Effects of HC on Extracellular Ca^2+^ Influx

To examine whether HC-induced vasorelaxation is mediated by Ca^2+^ channels, endothelium-denuded aortic rings were incubated in Ca^2+^-free Krebs solution for 45 min. The rings were then transferred to Ca^2+^-free KCl (80 mM) for 10 min to depolarize vascular smooth muscle cells and activate VOCCs. CaCl_2_ (10 µM to 10 mM) was subsequently added cumulatively to induce contraction, and responses were assessed in the presence and absence of HC (10^−6^, 10^−7^, and 10^−8^ mg/mL). Nifedipine, a calcium channel inhibitor, was used as a positive control. The percentage of contraction was compared between groups.

#### 4.5.5. The Effects of HC on Intracellular Ca^2+^ Release

To further investigate whether HC affects intracellular Ca^2+^ release from the SR in endothelium-denuded aortic rings, rings were first stimulated with KCl (80 mM) to promote Ca^2+^ release. They were then washed with Ca^2+^-free Krebs solution containing EGTA (1 mM) to prevent extracellular Ca^2+^ influx. Contraction was then induced with PE (10 µM) and caffeine (20 mM). For the contraction of the response curve, the rings were stimulated to contract twice before being incubated with HC (10^−6^, 10^−7^, and 10^−8^ mg/mL). The percentage of contraction was calculated and compared between conditions in the absence and presence of HC.

### 4.6. Statistical Analysis

Data were presented as mean ± S.E.M. Comparisons between two groups were conducted using an unpaired Student’s *t*-test. Multiple comparisons were analyzed using one-way analysis of variance (ANOVA), followed by Dunnett’s post hoc test. A *p*-value < 0.05 was considered statistically significant. Concentration-response curves were generated using non-linear regression in GraphPad Prism version 8 (GraphPad Software Inc., San Diego, CA, USA). HC induces vasorelaxation, which is partially mediated by the inhibition of both intra- and extracellular calcium influx. This includes the blockade of ROCCs and VOCCs. HC may also activate K_Ca_ channels, thereby promoting vascular dilation. These findings suggest that HC has the potential to induce vascular relaxation. Therefore, the results support further investigation of HC for the management of vascular dysfunction in cardiovascular disorders.

## Data Availability

The datasets generated and/or analyzed during the current study are available from the corresponding author upon reasonable request.

## Abbreviations

The following abbreviations are used in this manuscript:

HC	Hydrolyzed collagen
PE	Phenylephrine
KCl	Potassium chloride
TEA	Tetraethylammonium
BaCl_2_	Barium chloride
4-AP	4-aminopyridine
CaCl_2_	Calcium chloride
Ca^2+^	Calcium ion
K_Ca_	Calcium-activated potassium channel
Da	Dalton
ACE	Angiotensin-converting enzyme
MMP-9	Matrix Metalloproteinase-9
NF-κB	Nuclear Factor Kappa B
ACh	Acetylcholine
K^+^	Potassium ion
K_ATP_	ATP-sensitive potassium channel
K_V_	Voltage-dependent potassium channel
K_ir_	Inwardly rectifying potassium channel
VOCCs	Voltage-operated calcium channels
SR	Sarcoplasmic reticulum
ROCCs	Receptor-operated calcium channel
E_max_	Maximum Effect
cAMP	Cyclic adenosine monophosphate
L-type Ca^2+^	Long-lasting voltage-gated calcium
IP_3_R	Inositol 1,4,5-trisphosphate receptor
RyR	Ryanodine receptor
